# The clinical profile and associated mortality in people with and without diabetes with Coronavirus disease 2019 on admission to acute hospital services

**DOI:** 10.1002/edm2.309

**Published:** 2021-12-03

**Authors:** Krishna Gokhale, Samiul A. Mostafa, Jingya Wang, Abd A. Tahrani, Christopher Andrew Sainsbury, Konstantinos A. Toulis, G. Neil Thomas, Zaki Hassan‐Smith, Elizabeth Sapey, Suzy Gallier, Nicola Jaime Adderley, Parth Narendran, Srikanth Bellary, Tom Taverner, Sandip Ghosh, Krishnarajah Nirantharakumar, Wasim Hanif

**Affiliations:** ^1^ Institute of Applied Health Research University of Birmingham Birmingham UK; ^2^ School of Computer Science University of Birmingham Birmingham UK; ^3^ Midlands Health Data Research UK Birmingham UK; ^4^ Department of Diabetes Medicine University Hospitals of Birmingham Birmingham UK; ^5^ Institute of Metabolism and Systems Research University of Birmingham Birmingham UK; ^6^ Department of Diabetes and Endocrinology University Hospitals Birmingham NHS Foundation Trust Birmingham UK; ^7^ Institute of Cancer and Genomic Sciences University of Birmingham Birmingham UK; ^8^ Department of Endocrinology Queen Elizabeth Hospital Birmingham University Hospitals Birmingham NHS Foundation Trust Birmingham UK; ^9^ Centre for Endocrinology Diabetes and Metabolism Birmingham Health Partners Birmingham UK; ^10^ School of Life and Health Sciences Aston University Birmingham UK

**Keywords:** complications, COVID‐19, diabetes

## Abstract

**Introduction:**

To assess if in adults with COVID‐19, whether those with diabetes and complications (DM+C) present with a more severe clinical profile and if that relates to increased mortality, compared to those with diabetes with no complications (DM‐NC) and those without diabetes.

**Methods:**

Service‐level data was used from 996 adults with laboratory confirmed COVID‐19 who presented to the Queen Elizabeth Hospital Birmingham, UK, from March to June 2020. All individuals were categorized into DM+C, DM‐NC, and non‐diabetes groups. Physiological and laboratory measurements in the first 5 days after admission were collated and compared among groups. Cox proportional hazards regression models were used to evaluate associations between diabetes status and the risk of mortality.

**Results:**

Among the 996 individuals, 104 (10.4%) were DM+C, 295 (29.6%) DM‐NC and 597 (59.9%) non‐diabetes. There were 309 (31.0%) in‐hospital deaths documented, 40 (4.0% of total cohort) were DM+C, 99 (9.9%) DM‐NC and 170 (17.0%) non‐diabetes. Individuals with DM+C were more likely to present with high anion gap/metabolic acidosis, features of renal impairment, and low albumin/lymphocyte count than those with DM‐NC or those without diabetes. There was no significant difference in mortality rates among the groups: compared to individuals without diabetes, the adjusted HRs were 1.39 (95% CI 0.95–2.03, *p* = 0.093) and 1.18 (95% CI 0.90–1.54, *p* = 0.226) in DM+C and DM‐C, respectively.

**Conclusions:**

Those with COVID‐19 and DM+C presented with a more severe clinical and biochemical profile, but this did not associate with increased mortality in this study.

## Graphical abstract

- As people with diabetes are disproportionately affected by COVID‐19, there is a need to identify diabetes sub‐groups who could be at high risk of adverse outcomes when presenting to hospitals.
- Our real world study of people with diabetes and laboratory confirmed diagnoses of COVID‐19 uses service level data including biochemical markers for initial assessment. We compared these values and mortality levels in people with and without diabetes and hyperglycaemic complications.
- We found people with COVID‐19 and diabetes with existing complications presented with a more severe clinical and biochemical profile.
- However, the association between diabetic/hyperglycemic state in COVID‐19 and all cause‐mortality was not statistically significant in this study.

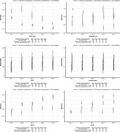

## 1 INTRODUCTION

Coronavirus disease 2019 (COVID‐19) has impacted on morbidity and mortality of people across the globe. People with diabetes mellitus (DM) represents one group particularly adversely affected.^1^ Recent studies have demonstrated people with DM have higher risks of more severe COVID‐19 outcomes, including higher mortality, as well as the presence of other co‐morbidities including cardiovascular disease (CVD) and chronic kidney disease (CKD).^2^, ^3^

Recent focus has been on in‐hospital morbidity and mortality. Within UK hospital settings, DM represents a significant proportion of all COVID‐19 cases and deaths.^3^, ^4^ In‐hospital mortality risks are increased in both type 1 and type 2 DM, however the former possesses the highest risk.^4^ As DM represents a highly prevalent and heterogeneous population, there is a need to identify additional DM sub‐groups who may be at higher risk of severe COVID‐19 related outcomes. One area for examination is the sub‐groups of DM with complications (DM+C) and DM with no complications (DM‐NC).

Secondly, whilst recognized COVID‐19 risk factors, including the presence of co‐morbidities, provide useful information on those at higher risk of acquiring COVID‐19, these risk factors do not account for initial clinical status or severity on initial admission to hospitals.^2^ A novel approach could be to examine service level hospital data, including blood tests and other measures, which clinicians use for initial patient assessment and progress. The results may aid strategies for early clinical risk stratification for treatment or alternatively, guide prevention strategies (*eg* vaccinations).

The aim of this study is to examine whether DM+C patients with COVID‐19 present with more adverse clinical and biochemical profiles and increased mortality compared to people with COVID‐19 and DM‐NC or without DM in an extensively phenotype adult cohort presenting to a large urban UK hospital.

## 2 METHODS

### 2.1 Study setting

This retrospective cohort study, using prospectively collected data, was conducted in Queen Elizabeth Hospital Birmingham (QEHB), a large teaching hospital within University Hospitals of Birmingham (UHB). It is situated in the city of Birmingham, West Midlands, UK and has over 1200 beds. West Midlands is a multi‐ethnic region with 30% of residents classified as Black, Asian and Minority Ethnic (BAME), of whom South Asians (18.9%) and Blacks (6.0%) are the most prevalent minorities.^5^ During the COVID‐19 crisis, patients were referred to QEHB from across the whole city and county due to high availability of hospital beds and specialist services. Patients with laboratory confirmed COVID‐19 were looked after by general internal medicine teams and cases were escalated to intensive care units for mechanical ventilation as per clinician judgement. Those with COVID‐19 and DM were assessed by a specialist diabetes team as needed. This study is reported as per the Reporting of studies Conducted using Observational Routinely‐collected health Data (RECORD) Statement.^6^

### 2.2 Data sources

We constructed the data using the Patient Administration Database (PAS) and the Electronic Medical Record system, known as the Patient Information and Communication System (PICS). The PAS database record information on age, gender, ethnicity, address (post code), primary reason for admission, discharge diagnostic codes, inpatient death, and discharge destination. Admission is defined as the time spent by an individual from recorded time of entry to recorded time of exit from the hospital. The PAS database was linked using unique patient identifiers (hospital number) to the PICS. It is a purpose‐designed system which records all in‐hospital prescriptions, laboratory results and electronic observations and generates alerts to reduce prescription errors and notify abnormal blood results.^7^ The linked PAS‐PICS databases have been used for multiple diabetes related research.^8^, ^9^, ^10^

### 2.3 Study population

All adult patients (>16 years old) who presented to QEHB from 20th of March to 9th of June 2020 with a confirmed positive swab specimen result for COVID‐19 were included in the analysis.

### 2.4 Data collection and variable definitions

Patient demographics and clinical data were collected from PAS and PICS. Clinician confirmed co‐morbidities were available from PAS and PICS, complemented by in‐hospital prescription data and diagnostic codes derived from previous hospital admissions. The PICS encodes diagnoses using NHS Digital SNOMED CT browser alongside and mapped on to ICD‐10 codes allowing for the presentation and inclusion of historically entered ICD‐10 codes. The composite CVD, hypertension, severe renal diseases, dementia, chronic obstructive pulmonary disease, cancer, asthma, atrial fibrillation, were defined by the combination of ICD‐10 codes and PAS‐PICS encoded diagnoses, whichever was available at study entry. The composite CVD was defined as one of the following presentations: myocardial infarction, peripheral vascular disease, heart failure, cerebrovascular infarction, stroke, transient ischaemic attack and ischaemic heart disease.

### 2.5 Clinical assessments

All patients underwent nasopharyngeal and oropharyngeal swab specimen (miniature absorbent pads) testing for COVID‐19. These were processed in accordance with NHS guidance within UHB NHS laboratories.^11^ The swab specimens were measured for COVID‐19 using either real‐time reverse transcription polymerase chain reaction or transcription mediated amplification methods on one of three assays: Abbott M2000, Cepheid GeneXpert or a Hologic Panther. Co‐efficient of variation values were based on calibrations and therefore varied between individual runs.

Venepuncture was conducted to ascertain venous blood for routine metabolic blood tests; the first blood tests were taken before administration of any intravenous fluids. An arterial blood gas was performed to assess for acid‐base status and estimated partial pressure of oxygen and carbon dioxide levels. Physiological assessments included measurement of respiratory rate and pulse rate via a pulse auxometer, systolic and diastolic blood pressures and temperature. All swab specimens, blood tests and physiological assessments were performed by trained healthcare professionals following standard operating procedures.

All physiological and laboratory measurements were categorized based on clinically meaningful thresholds and the earliest available measurement was used in the analysis. Missing data were presented as a missing category for all measurements.

### 2.6 Definitions of DM, DM+C and glycaemic categories for analysis

DM was defined as those who with an ICD‐10 record of DM or its complications, or who were recorded to have been prescribed any of the DM drugs using a previously published algorithm.^9^ DM complication status was determined by the record of ICD‐10 Read codes for DM complications, which mainly included diabetes microvascular complications (ophthalmic complications, neurological complications, renal complications, and peripheral circulatory complications). Based on the DM and complications status, patients were categorized into three groups: (1) people with DM and microvascular complications (DM+C), (2) people with DM but no microvascular complications (DM‐NC) and (3) people without DM (Non‐diabetes).

### 2.7 Covariates

Ethnicity was self‐reported by the patient or their family members on admission to hospital. Body mass index (BMI) was categorized based on the World Health Organisation Criteria: normal weight (BMI of < 25 kg/m^2^), overweight (BMI of 25 kg/m^2^ to < 30 kg/m^2^), obesity (BMI of 30 kg/m^2^ to < 35 kg/m^2^), obesity II & III (BMI of ≥35 kg/m^2^). Charlson Comorbidity Index (CCI) was calculated (DM and DM complication score were removed from the equation) using ICD‐10 code and was categorized into four groups (0, 1, 2, and ≥3).^12^

In the non‐diabetes, DM‐NC and DM+C groups, we looked at the trends of available physiological and laboratory measurements in the first 5 days after admission. These included measures of metabolic acidosis and compensation (anion gap, partial pressure of carbon dioxide (pCO2), bicarbonate (HCO3‐) and hydrogen ions), indicators of underlying presence of inflammation (serum C‐reactive protein, CRP), measures of immune response (lymphocyte count), serum electrolytes and renal function (Na+, K+, urea, estimated glomerular filtration rate, eGFR) and other clinically useful physiological and laboratory measurements (partial pressure of oxygen (pO_2_), heart rate, temperature and serum albumin). All these measurement across three groups are presented visually as mean (standard error, SE) or median (IQR) for symmetrical and skewed continuous variables, respectively.

### 2.8 Follow‐up and outcome

All eligible patients were followed‐up from hospital admission until the earliest of any censoring event (patient discharged, death, study end date) in hours. A small proportion (3.2%) of patients were not discharged at study end‐date.

### 2.9 Statistical analyses

Baseline characteristics for the total population and DM sub‐groups are presented as mean ± standard deviation (standard deviation, SD) or median (interquartile range, IQR) for symmetrical/ skewed continuous variables and as frequency (percentage) for categorical variables. All physiological and laboratory measurements were compared across non‐diabetes, DM‐NC, and DM+C groups using ANOVA or Kruskal‐Wallis test depending on data distribution. For categorical variable comparisons, Chi‐square test was applied.

### 2.10 Regression analysis

Cox proportional hazard regression models were used to calculate crude and adjusted hazard ratios (aHRs), together with their corresponding 95% Confidence intervals (CI). Covariates in the Cox model for mortality included age, sex, ethnicity, BMI categories, and CCI categories (model comprised the interaction effect between age and CCI categories). Missing data for BMI and ethnicity were included in the Cox model as a missing category. All statistical tests were two‐tailed and a p‐value < 0.05 was considered statistically significant. All analyses were conducted in R 4.0.0 (The R Foundation for Statistical Computing).

## 3 RESULTS

### 3.1 Baseline characteristics

There were 996 people with a laboratory confirmed positive swab specimen for COVID‐19 admitted to QEHB from 20th March to 9th June 2020. In the total cohort, the mean age was 68 years. Among them 399 (40%) had DM, of whom 104 and 295 patients had codes indicative of DM+C and DM‐NC, respectively, Table 1. Compared to people without diabetes, people with DM+C or DM‐NC were more likely to be men, from South Asian background and have obesity. People with DM+C had the highest levels of obesity, CCI score ≥3, CVD, ischaemic heart disease, stroke/TIA, heart failure, hypertension and end‐stage renal disease (ESRD). Levels of other co‐morbidities, including dementia, cancer, COPD, asthma and AF, did not vary between the three sub‐groups.

**TABLE 1:** Baseline demographic characteristics and co‐morbidities of the COVID‐19 cohort, stratified by glycaemic and complication status

	Overall	Non‐diabetes	DM‐NC	DM+C	*p*‐value
N	996	597	295	104	
Age (years)	68.4 ± 17.5	68.1 ± 19.0	68.1± 15.2	70.6 ± 13.9	.397
Male	559 (56.1)	314 (52.6)	184 (62.4)	61 (58.7)	.019
Ethnicity					
White	568 (57.0)	382 (64.0)	140 (47.5)	46 (44.2)	<.001
South Asian	149 (15.0)	60 (10.1)	62 (21.0)	27 (26.0)	
Black	63 (6.3)	25 (4.2)	24 (8.1)	14 (13.5)	
Others	48 (4.8)	26 (4.4)	18 (6.1)	4 (3.8)	
Mixed	11 (1.1)	6 (1.0)	2 (0.7)	3 (2.9)	
Missing	157 (15.8)	98 (16.4)	49 (16.6)	10 (9.6)	
BMI (kg/m^2^)	29.1 ± 7.7	28.0 ± 8.0	30.3 ± 6.9	32.0 ± 7.1	<.001
BMI categories					
<25	282 (28.3)	200 (33.5)	66 (22.4)	16 (15.4)	<.001
25 to < 30	327 (32.8)	201 (33.7)	95 (32.2)	31 (29.8)	
30 to < 35	182 (18.3)	99 (16.6)	59 (20.0)	24 (23.1)	
≥35	172 (17.3)	72 (12.1)	67 (22.7)	33 (31.7)	
Missing	33 (3.3)	25 (4.2)	8 (2.7)	0 (0.0)	
Charlson co‐morbidity index					
0	212 (21.3)	145 (24.3)	62 (21.0)	5 (4.8)	<.001
1	196 (19.7)	128 (21.4)	62 (21.0)	6 (5.8)	
2	131 (13.2)	89 (14.9)	36 (12.2)	6 (5.8)	
≥3	457 (45.9)	235 (39.4)	135 (45.8)	87 (83.7)	
Cardiovascular diseases	431 (43.3)	216 (36.2)	140 (47.5)	75 (72.1)	<.001
Ischaemic heart disease	228 (22.9)	99 (16.6)	80 (27.1)	49 (47.1)	<.001
Stroke/TIA	78 (7.8)	36 (6.0)	26 (8.8)	16 (15.4)	.004
Heart failure	163 (16.4)	77 (12.9)	52 (17.6)	34 (32.7)	<.001
Hypertension	628 (63.1)	305 (51.1)	227 (76.9)	96 (92.3)	<.001
End‐Stage Renal Disease	100 (10.0)	28 (4.7)	23 (7.8)	49 (47.1)	<.001
Dementia	341 (34.2)	203 (34.0)	97 (32.9)	41 (39.4)	.473
COPD	260 (26.1)	159 (26.6)	79 (26.8)	22 (21.2)	.478
Cancer	125 (12.6)	82 (13.7)	30 (10.2)	13 (12.5)	.319
Asthma	153 (15.4)	88 (14.7)	49 (16.6)	16 (15.4)	.767
Atrial Fibrillation	228 (22.9)	130 (21.8)	71 (24.1)	27 (26.0)	.547

Note

Data were presented as mean ± SD or n (%). DM‐NC: Patients with diabetes mellitus but no complications; DM+C: Patients with diabetes mellitus and complications.

Abbreviations: BMI, body mass index; COPD: Chronic obstructive pulmonary disease. P‐values are for comparisons between the three sub‐groups, ANOVA was used for mean comparison, Kruskal‐Wallis test was used for median comparison; TIA, Transient ischaemic attack.

### 3.2 Physiological and laboratory measurements at presentation and in the first 5 days

Individuals with DM+C were more likely to present with a pH level < 7.3 and a higher anion gap than in those with DM‐NC or those without DM, *p* = .001 and *p* < .001 respectively (Table 2). Features of renal impairment (high urea, raised K+ and lower eGFR) were more common at presentation in the DM+C group than in the DM‐NC group or those without DM, which could be related to underlying CKD, dehydration or acute kidney injury associated with acute COVID‐19. In particular, eGFR < 30ml/min/1.73m^2^ was more common in people with DM+C (54.8%) than those with DM‐NC (18.6%) and those without DM (12.9%), *p* < .001. People with DM+C also had lower serum albumin and lymphocyte count.

**TABLE 2:** Baseline clinical and laboratory test measurements according to glycaemic and complication status

	Non‐diabetes	DM‐NC	DM+C	p*
N	597	295	104	
Anion Gap (mmol/l)	19.1 ± 3.2	19.5 ± 3.8	21.7 ± 5.9	<.001
Anion Gap categories				
6 to < 16	51 (8.5)	27 (9.2)	3 (2.9)	
≥16	324 (54.3)	175 (59.3)	57 (54.8)	.169
Missing	222 (37.2)	93 (31.5)	44 (42.3)	
pCO_2_ (kPa)	5.5 ± 1.3	5.6 ± 1.4	5.5 ± 1.2	.285
pCO_2_ categories				
<4.67	110 (18.4)	53 (18.0)	17 (16.3)	
4.67 to <6.4	202 (33.8)	114 (38.6)	44 (42.3)	.362
≥6.4	86 (14.4)	58 (19.7)	25 (24.0)	
Missing	199 (33.3)	70 (23.7)	18 (17.3)	
HCO_3_‐ (mmol/l)	24.9 ± 4.4	24.3 ± 4.8	23.8 ± 5.6	.105
HCO_3_‐ categories				
<22	88 (14.7)	65 (22.0)	25 (24.0)	
22 to <29	252 (42.2)	130 (44.1)	47 (45.2)	.251
≥29	54 (9.0)	27 (9.2)	14 (13.5)	
Missing	203 (34.0)	73 (24.7)	18 (17.3)	
pH	7.41 ± 0.07	7.38 ± 0.07	7.37 ± 0.10	<.001
pH categories				
<7.30	23 (3.9)	22 (7.5)	14 (13.5)	
7.30 to <7.35	35 (5.9)	35 (11.9)	12 (11.5)	.001
7.35 to <7.45	233 (39.0)	128 (43.4)	45 (43.3)	
≥7.45	103 (17.3)	37 (12.5)	15 (14.4)	
Missing	203 (34.0)	73 (24.7)	18 (17.3)	
Urea (mmol/l)	6.2 (4.3 – 10.5)	7.2 (5.0 – 12.4)	12.9 (8.2 – 19.9)	0
Urea categories				
<7.8	361 (60.5)	155 (52.5)	20 (19.2)	<.001
≥7.8	208 (34.8)	128 (43.4)	80 (76.9)	
Missing	28 (4.7)	12 (4.1)	4 (3.8)	
Potassium (K+, mmol/l)	4.1 ± 0.5	4.3 ± 0.7	4.6 ± 0.8	<.001
K categories				
2.5 to <5.3	518 (86.8)	239 (81.0)	73 (70.2)	<.001
≥5.3	13 (2.2)	19 (6.4)	14 (13.5)	
Missing	66 (11.1)	37 (12.5)	17 (16.3)	
Sodium (Na+, mmol/l)	138.7 ± 6.5	137.2 ± 6.7	136.9 ± 6.8	.001
Na categories				
<133	62 (10.4)	58 (19.7)	14 (13.5)	.002
133 to <145	435 (72.9)	199 (67.5)	79 (76.0)	
≥145	72 (12.1)	26 (8.8)	7 (6.7)	
Missing	28 (4.7)	12 (4.1)	4 (3.8)	
eGFR (ml/min/1.73m^2^)	73 (47 – 90)	63 (39 – 88)	22 (8 – 47)	0
eGFR categories				
<30	77 (12.9)	55 (18.6)	57 (54.8)	
30 to <60	119 (19.9)	72 (24.4)	24 (23.1)	<.001
≥60	372 (62.3)	156 (52.9)	18 (17.3)	
Missing	29 (4.9)	12 (4.1)	5 (4.8)	
Lymphocytes (x 10^9^/L)	1.0 (0.7 – 1.3)	0.9 (0.6 – 1.4)	0.8 (0.6 – 1.0)	.006
Lymphocytes categories				
<1.5	465 (77.9)	220 (74.6)	89 (85.6)	.052
≥1.5	103 (17.3)	64 (21.7)	12 (11.5)	
Missing	29 (4.9)	11 (3.7)	3 (2.9)	
CRP (mg/L)	99 (41 – 168)	113.5 (55.5 – 192.8)	89 (43 – 137)	.023
CRP categories				
<10	47 (7.9)	19 (6.4)	6 (5.8)	
10 to < 100	233 (39.0)	104 (35.3)	50 (48.1)	.139
≥100	277 (46.4)	153 (51.9)	41 (39.4)	
Missing	40 (6.7)	19 (6.4)	7 (6.7)	
Albumin (g/L)	30.2 ± 6.1	29.0 ± 6.0	28.1 ± 6.3	.001
Albumin categories				
<25	95 (15.9)	62 (21.0)	26 (25.0)	
25 to < 35	326 (54.6)	167 (56.6)	55 (52.9)	.07
≥35	130 (21.8)	50 (16.9)	17 (16.3)	
Missing	46 (7.7)	16 (5.4)	6 (5.8)	
Temperature (Celsius)	36.9 ± 1.0	37.0 ± 1.2	36.7 ± 1.2	.031
Temperature categories				
<37.8	489 (81.9)	218 (73.9)	84 (80.8)	.022
≥37.8	107 (17.9)	76 (25.8)	20 (19.2)	
Missing	1 (0.2)	1 (0.3)	0 (0.0)	
Heart rate (beats/min)	91.3 ± 19.6	94.2 ± 19.9	86.5 ± 19.0	.002
Heart rate categories				
<80	161 (27.0)	69 (23.4)	39 (37.5)	
80 to <100	267 (44.7)	114 (38.6)	44 (42.3)	.002
≥100	168 (28.1)	111 (37.6)	21 (20.2)	
Missing	1 (0.2)	1 (0.3)	0 (0.0)	
pO_2_ (kPa)	6.4 (3.9 – 9.5)	6.9 (4.1 – 9.8)	5.5 (3.7 – 8.3)	.209
pO_2_ categories				
<7.3	234 (39.2)	117 (39.7)	57 (54.8)	
7.3 to <10	74 (12.4)	53 (18.0)	11 (10.6)	.147
≥10	90 (15.1)	54 (18.3)	18 (17.3)	
Missing	199 (33.3)	71 (24.1)	18 (17.3)	

Note

Data were presented as Mean ±SD, Median (25^th^ – 75^th^), or n (%). DM‐NC: Patients with diabetes mellitus but no complications; DM+C: Patients with diabetes mellitus and complications.

*All *p*‐values for categorical variables were calculated without ‘Missing’ category, ANOVA was used for mean comparison, Kruskal‐Wallis test was used for median comparison.

Abbreviations: CRP, C‐reactive protein; eGFR, estimated glomerular filtration rate; HCO3‐, bicarbonate; pCO2, partial pressure of carbon dioxide.

People with DM+C had lower levels of serum CRP, heart rate or temperature compared to people with DM‐NC and those without DM.

**FIGURE 1:**
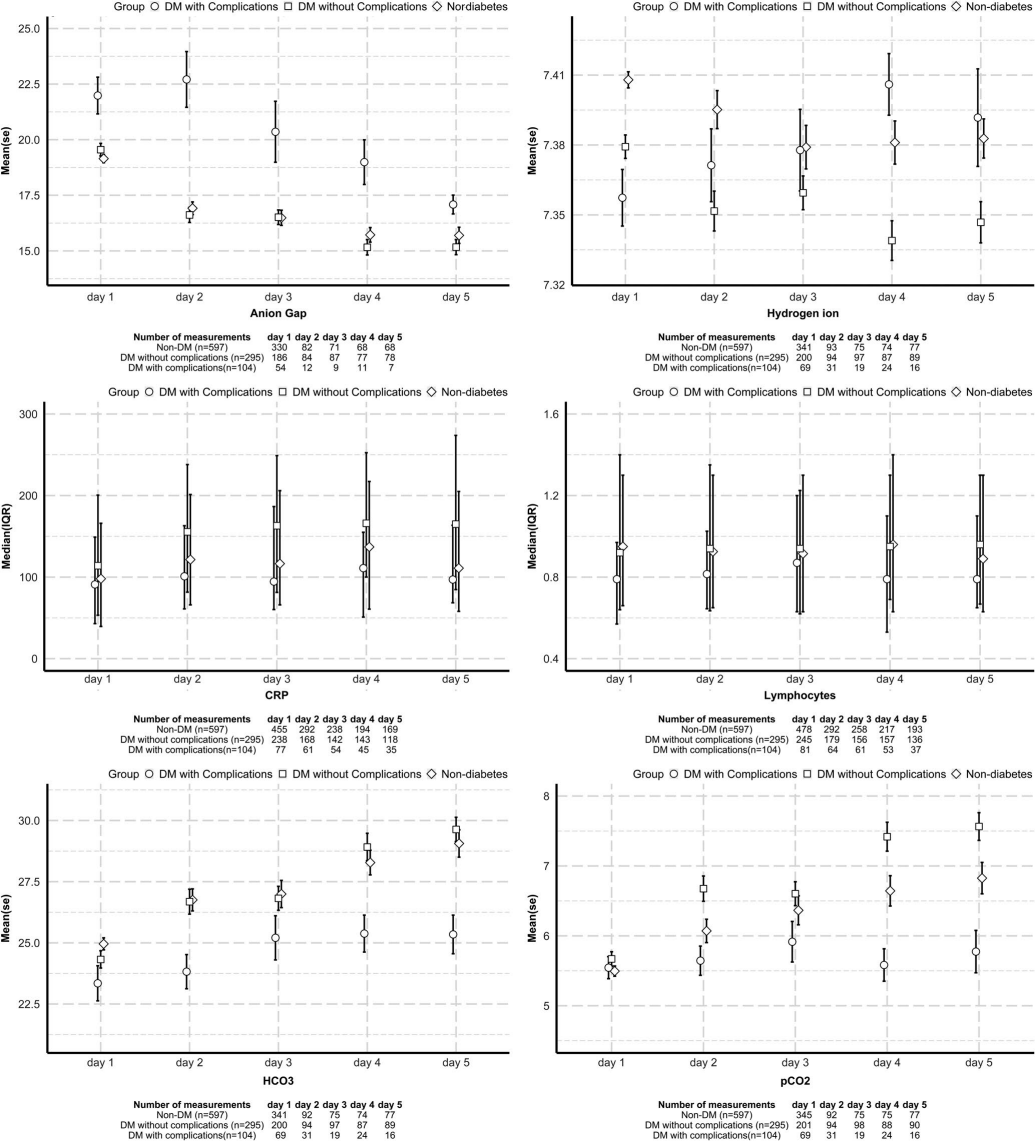
Measures of metabolic acidosis, I inflammation and immune response after hospital admission. Data presented for mean or median values over time. Key: HCO_3_, bicarbonate level; pCO_2_, partial pressure of carbon dioxide; K, potassium level; CRP, C‐reactive protein level; Na, sodium level; Ur, urea level; pO_2_, partial pressure of oxygen

**FIGURE 2:**
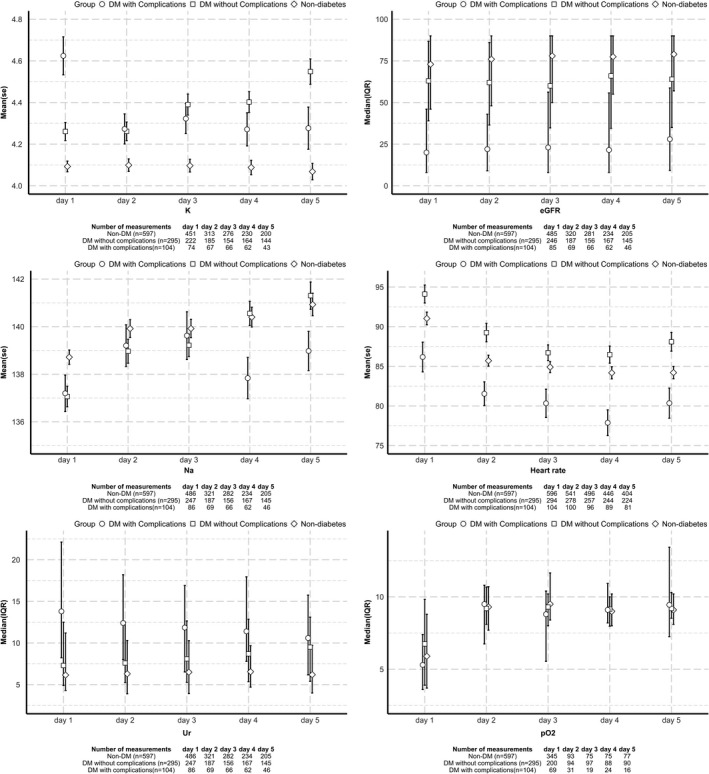
Renal function, electrolytes, and physiological and laboratory measurements after hospital admission. Data presented for mean or median values over time. Key: HCO_3_, bicarbonate level; pCO_2_, partial pressure of carbon dioxide; K, potassium level; CRP, C‐reactive protein level; Na, sodium level; Ur, urea level; pO_2_, partial pressure of oxygen

Where measurements were available, these observations largely persisted in the first 5 days after admission (Figures 1&2).

### 3.3 Mortality rates

There were 309 in‐hospital deaths during follow‐up: 40 (38%) in patients with DM+C, 99 (34%) in people with DM‐NC and 170 (28%) in people without DM (Table 3). Patients with DM were 23% more likely to die in comparison to patients without DM after adjusting for age, sex, ethnicity, BMI and CCI: aHR 1.23 (95% CI 0.96, 1.57).

**TABLE 3:** Risk of COVID‐19 associated mortality according to glycaemic and complication status

Groups	Non‐diabetes	DN‐NC	DM+C
Total number of patients	597	295	104
Number of outcomes	170	99	40
Person‐days (pd)	172,720	107,880	28,533
Follow‐up days	173 (82 – 382)	233 (116 – 513)	180 (116 – 321)
Incidence rate per 1000 pd	0.98	0.92	1.40
Crude HR (95% CI)	1.00 (1.00, 1.00)	0.96 (0.75, 1.24)	1.37 (0.97, 1.94)
P‐value for Crude HR	‐	0.776	0.071
Adjusted HR (95% CI)^†^	1.00 (1.00, 1.00)	1.18 (0.90, 1.54)	1.39 (0.95, 2.03)
P‐value for Adjusted HR	‐	0.226	0.093

Abbreviations: CI: confidence intervals; DM‐NC: Patients with diabetes mellitus but no complications; DM+C: Patients with diabetes mellitus and complications; HR, hazard ratios, pd: person days. P‐values compared to Non‐diabetes.

† Adjusted for age, sex, ethnicity, BMI categories, and Charlson Comorbidity Index categories (model comprised the interaction effect between age and Charlson Comorbidity Index categories).

The mortality rate was higher in patients with DM+C (aHR 1.39, 95% CI 0.95, 2.03) and in those with DM‐NC (1.18, 95% CI 0.90, 1.54) compared to patients without diabetes. However, these findings did not reach statistical significance, p‐values of 0.093 and 0.226 respectively.

## 4 DISCUSSION

Our study shows that people admitted with symptomatic COVID‐19 and DM were more likely to be men, from a BAME background, and had higher BMI and more CVD, and more ESRD compared to those without DM. In addition, patients with DM+C had higher BMI, CVD and more ESRD compared to DM‐NC, as would have been expected. Patients with DM+C had higher anion gap, urea, potassium, and lower pH, lymphocytes, albumin, compared to DM‐NC. In addition, the DM+C group had lower heart rate, higher BP, less tachypnoea, lower Hb compared to patients with DM‐NC. Patients in the DM+C group had a 39% higher mortality rates than people without DM or with DM‐NC, but this did not reach significance. Other predictors of higher mortality included age, higher CCI, men and BAME groups.

The relatively higher mortality observed in people with DM compared to those without DM in this study is consistent with that reported previously in other COVID‐19 studies and other studies showing higher mortality in patients with DM in relation to influenza, SARS and MERS.^13^, ^14^ This increases risk of adverse outcomes from these viral infections is likely due to multiple mechanisms including impaired immune response within a hyperglycaemic environment and reduced cellular expression of angiotensin‐converting enzyme (ACE) 2, leaving cells prone to damage through inflammation.^14^, ^15^, ^16^

The mortality risk was non‐significantly greater in people with DM+C than DM‐NC or those without DM. This in part could be due to differences in BMI, ethnicity, CCI, CVD and ESRD, which have been reported previously to be associated with increased risk of adverse COVID‐19 outcomes.^2^. Diabetes autonomic neuropathy (DAN), which is common in people with DM, especially in the presence of complications, might also contribute to the increased mortality considering the established associations between DAN, CVD, CKD and mortality in DM.^17^, ^18^, ^19^ This is supported by the results showing differences in heart rate and respiratory rate between patients with DM+C and DM‐NC. In addition, patients with DM+C had biochemical features to suggest more hypovolaemia/ dehydration on admission (higher urea, higher anion gap and lower pH level), which might be caused either by having more severe infection or the presence of underlying complications such as renal impairment and DAN. However, these parameters remained in the normal defined range and had a relatively wide standard deviation.

DM+C is usually associated with longer duration of DM and more adverse glycaemic control, which in turn may impact on pathological mechanisms affecting the response to COVID‐19. Furthermore, the hyperglycaemic complication itself could leave the body more prone to a more adverse outcome where it impacts on ACE‐2 cell expression. A recent study found worse glycaemic control was a risk factor for increased mortality.^3^

The results of this study need to be considered in the context of its limitations. This study was conducted from a single centre which might affect the external validity of the findings. However, this single centre is a large, tertiary and receives patients form a large population beyond its localities, especially during the COVID‐19 crisis. The sample size of our study is relatively small, as was the follow‐up period of three months, which was reflected in the some of the 95% CIs reported in the study, and is reflected by the 39% increased risk of mortality in COVID‐19 DM+C patients compared to non‐diabetes not reaching significance. For the same reason, we were unable to analyse results and outcomes between those with type 1 and 2 diabetes. Secondly, the results reflect the findings in people who attended the hospital but does not account for COVID‐19 cases treated in the community setting only. Furthermore, some variables had missing data, but to minimize the impact of this on the results, missing data categories were used in the multivariable analysis. We were unable to account for some variables including use of medications for diabetes or hypertension. Also it was not possible to account for people with pre‐diabetes in this study. Finally, data regarding HbA1c and diabetes duration were not available in our analysis, although those with complications are likely to have higher HbA1c and longer diabetes duration.

The strengths of this study include the in‐depth phenotyping which was made possible with the presence of the appropriate data management systems in the hospital trust. Also, cases of COVID‐19 were laboratory confirmed. In addition, our study population included multiple ethnicities and in proportions mirroring those of the West Midlands county.^5^

Finally, a recent mortality risk score has been developed for the general population, which utilizes total number of co‐morbidities as one parameter for scoring, without considering presence/ absence of diabetes separately.^20^ During development of the risk score, biochemical test variables assessed for inclusion did not include measurements of acid‐base status including pH, bicarbonate or anion gap. It is not known if this could add further benefits to a risk score.

## 5 CONCLUSION

In this multi‐ethnic cohort of adults with COVID‐19 presenting to hospitals, we found clinical and biochemical profiles were adverse in people with DM+C.

## CONFLICT OF INTEREST

SAM has received research support from Novo Nordisk Research Foundation UK and Academy of medical Sciences. SB has received grants, personal fees, and support to attend educational meetings from Novo Nordisk; grants from The Binding Site; personal fees from AstraZeneca, Merck, Sharpe & Dohme, and Janssen; personal fees and support to attend educational meetings from Boehringer Ingelheim; personal fees and support to attend educational meetings from Eli Lilly; and personal fees and support to attend educational meetings from Sanofi‐Aventis and NAPP. WH has received Received Research Grants, Travel Grant and Consultancy for following AZ, BI, Eli Lilly, Jansen, Novo Nordisk, Sanofi, NAPP and MSD. Other authors have no declarations.

## AUTHOR CONTRIBUTIONS

**Krishna Gokhale:** Software (equal); Writing‐original draft (equal). **Jingya Wang:** Investigation (equal); Validation (equal); Writing‐review & editing (equal). **Abd A. Tahrani:** Writing‐review & editing (equal). **G. Neil Thomas:** Writing‐review & editing (equal).

## Data Availability

Research data are not shared.
